# Sheathless CE-MS based metabolic profiling of kidney tissue section samples from a mouse model of Polycystic Kidney Disease

**DOI:** 10.1038/s41598-018-37512-8

**Published:** 2019-01-28

**Authors:** Elena Sánchez-López, Guinevere S. M. Kammeijer, Antonio L. Crego, María Luisa Marina, Rawi Ramautar, Dorien J. M. Peters, Oleg A. Mayboroda

**Affiliations:** 1 Department of Analytical Chemistry, Physical Chemistry and Chemical Engineering, University of Alcalá, Madrid, Spain; 2 Center for Proteomics and Metabolomics, Leiden University Medical Center, Leiden, The Netherlands; 3 Biomedical Microscale Analytics, Leiden Academic Center for Drug Research, Leiden University, Leiden, The Netherlands; 4 Department of Human Genetics, Leiden University Medical Center, Leiden, The Netherlands

## Abstract

Capillary electrophoresis-mass spectrometry (CE-MS) using a sheathless porous tip interface emerged as an attractive tool in metabolomics thanks to its numerous advantages. One of the main advantages compared to the classical co-axial sheath liquid interface is the increased sensitivity, while maintaining the inherent properties of CE, such as a high separation efficiency and low sample consumption. Specially, the ability to perform nanoliter-based injections from only a few microliters of material in the sample vial makes sheathless CE-MS a well-suited and unique approach for highly sensitive metabolic profiling of limited sample amounts. Therefore, in this work, we demonstrate the utility of sheathless CE-MS for metabolic profiling of biomass-restricted samples, namely for 20 µm-thick tissue sections of kidney from a mouse model of polycystic kidney disease (PKD). The extraction method was designed in such a way to keep a minimum sample-volume in the injection vial, thereby still allowing multiple nanoliter injections for repeatability studies. The developed strategy enabled to differentiate between different stages of PKD and as well changes in a variety of different metabolites could be annotated over experimental groups. These metabolites include carnitine, glutamine, creatine, betaine and creatinine. Overall, this study shows the utility of sheathless CE-MS for biomass-limited metabolomics studies.

## Introduction

Polycystic kidney disease (PKD) is a complex clinical entity unifying a group of diseases that results in renal cyst development^1^. The animal models and the rodents in particular are essential for the understanding of the biochemical mechanisms of the disease, providing information which would be impossible to get from the observational human studies only. The trend towards a more responsible use of the laboratory animals stimulates the changes in analytical methodologies demanding analytical workflows for the volume restricted biological samples. Metabolomics is one of the areas where such methods are highly needed. For instance, the onset and progression of autosomal dominant polycystic kidney disease (ADPKD) is commonly explained as an effect of the dysregulated expression of the *PKD1* or *PKD2* genes. The encoded proteins are associated with remodeling of the tubular architecture and as such, directly or indirectly, control multiple signaling cascades in renal epithelial cell. This, in turn, leads to a profound metabolic remodeling of the affected cells. Consequently, it raises a question whether such remodeling contributes to the PKD pathophysiology^2,3^. Here, we would like to address this question using kidney-specific PKD1-deletion mice, of which we analysed single 20 μm tissue sections.

There are different technical solutions available, but here we are presenting a report on using capillary electrophoresis – mass spectrometry (CE-MS) for metabolic profiling of such material. CE-MS offers many advantages that makes it a useful tool in metabolomics studies. One can mention minimal sample requirement (usually only a few nanoliters are injected) or the high separation efficiency due to the flat flow profile of the electro-osmotic flow where only longitudinal diffusion is contributing to band broadening in CE^4^. Moreover, the method has a proven track record for the analysis of polar and charged compounds^5^. However, as every analytical technique, CE-MS has its weaknesses. Limitations of CE include low migration time repeatability and relatively poor detection sensitivity due to the low injection volume. Moreover, in the case of employing a sheath-liquid interface an extra dilution occurs thereby compromising the sensitivity even further^4^. In the past years, significant developments addressed these limitations and resulted in improved interfacing techniques for CE-MS among them the sheathless porous tip interface^6^. The porous tip interface (offered by SCIEX as CESI sprayer) can substantially improve the detection sensitivity (i.e., up to 100-fold) as compared to using a conventional co-axial sheath-liquid interface^7,8^. Another advantage of the porous tip sprayer interface is reduced ion suppression, which is inherent to the use of flow rates below 20 nL/min in ESI-MS^9^. For a variety of reasons, the potential of CESI-MS for metabolomics has hardly been explored. Only a limited number of studies were reported using urine^7,10^, plasma^11,12^, CSF^11^, and cells^13–16^. Thus, with this report we demonstrate the utility of CESI-MS for metabolic profiling of biomass-restricted tissue material and, at the same time, we provide a descriptive study of the metabolic changes associated with PKD progression^1^.

## Results and Discussion

For decades, laboratory animals and rodents in particular are being used as the main experimental tool of the modern biology. They offer a unique possibility for modeling the human diseases *in vivo*, getting insight into the systemic and local effects of a given pathology. Miniaturization of the analytical workflows is one of the most obvious demands. With regard to metabolomics the diversity of the mammalian metabolome implies availability of several micro-scale strategies which could be adopted to a particular class of the metabolites or the sample types. CE-MS has a proven track record as a micro-scale method, known for its possibility to provide complex biological profiles from only a limited amount of material. Here, we demonstrate the feasibility of this approach for an animal model for PKD using a specific type of biomass-restricted samples, namely 20 µm-thick tissue sections of mouse kidneys. The set of the samples consisted of four groups. The two wild type groups (Wt) at the beginning of the experiment and 14 weeks into the experiment (Wt0 and Wt14). The other two groups represent a mild (MCK) and advanced (ESCK) kidney damage conditions.

### Potential of Sheathless CE-MS for Metabolic Profiling of Biomass-Restricted Samples

**Figure 1:**
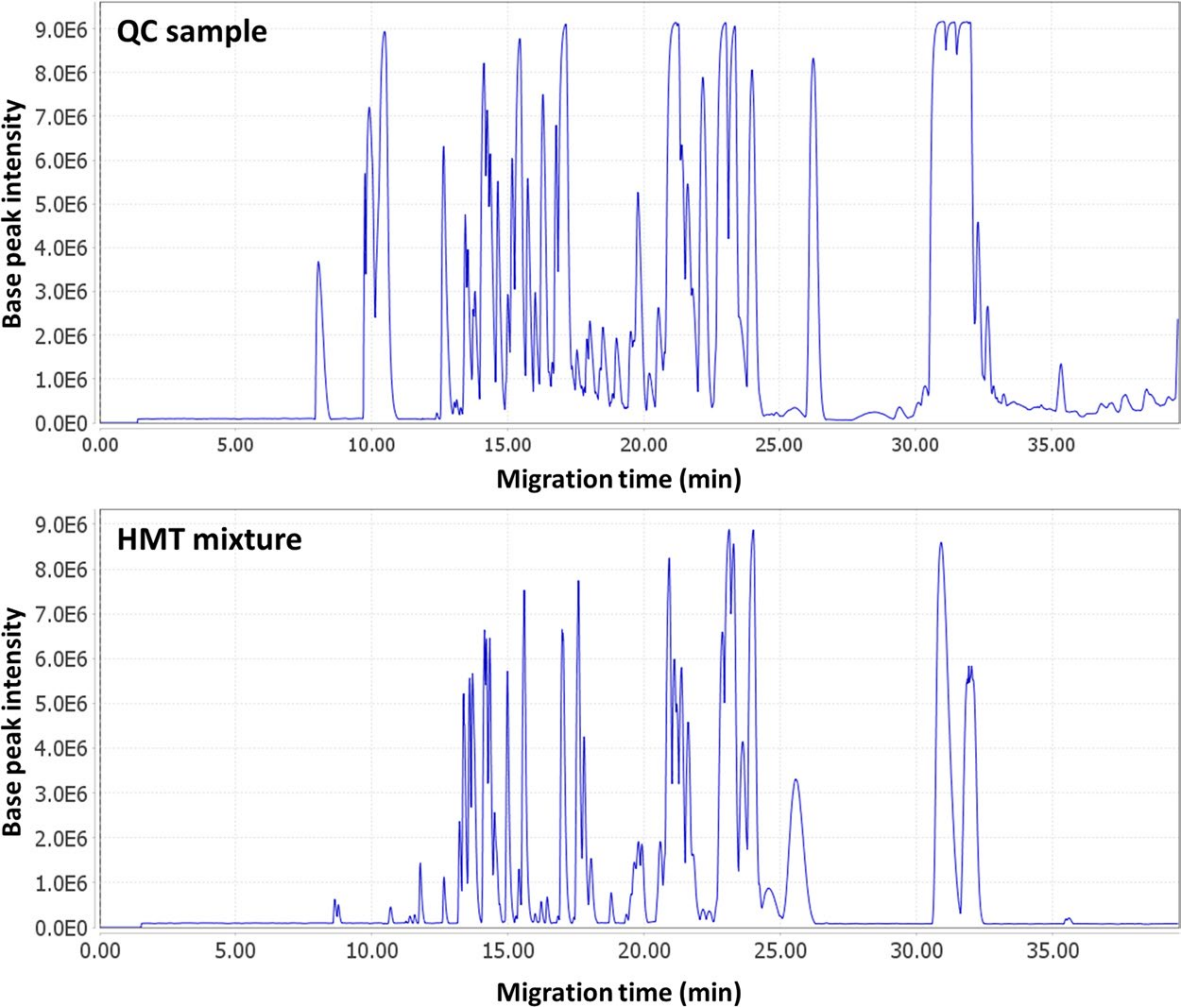
Comparison of base peak electropherograms of standard metabolite mixture consisting of 60 metabolites at 50 µM (**A**) and a QC sample of tissue sections (**B**) obtained with the sheathless CE-MS platform. Experimental conditions: BGE, 10% (v/v) acetic acid (pH 2.3); voltage, +20 kV; sample injection, 1 psi × 60 s (1.4% total volume of the capillary, i.e. roughly 9 nL); 91 cm × 30 µm i.d. × 150 µm o.d. fused-silica capillary.

There are several technical solutions for integration of CE and MS^6,15,17–19^. Here, we use a CESI-MS platform, a sheathless CE-MS configuration, where CE separation and ESI ionization work as a single process^8^. Moreover, following the recent work of Kammeijer *et al*.^20^ in which a dopant-enriched nitrogen gas (DEN-gas) was employed to further increase the detection sensitivity of CESI-MS, we also used the DEN-gas attenuated version of CESI. To test the general performance of this approach as a tool for metabolic profiling, standard metabolite mixtures consisting of sixty compounds in an equimolar concentration (HMT mixture) have been analyzed. This mixture includes the major metabolite classes such as amino acids, betaines, lactams, purines and nucleosides. Figure 1 shows a base peak electropherogram (BPE) obtained by CESI-MS for the standard metabolite mixture. It is important to note that it was possible to obtain low nanomolar detection limits for most metabolites in this standard mixture, this being in agreement with previous CESI-MS metabolomics studies^7^. The second Figure in the panel (Fig. 1) shows the BPE of a quality control (QC) sample, i.e. a pooled sample containing equally volumes of all samples used in this study. Not surprisingly, the complexity of the QC sample exceeds the one of the standard metabolite mixture. Moreover, despite a general pattern similarity, there is a clear difference between the metabolite mixture and QC sample in the responses observed for individual metabolites. Supplementary Fig. S-1 clearly illustrates this point by showing extracted ion electropherograms for various selected metabolites from the different chemical families.

A detailed description of the analytical sequence is given in section 4.5. Apart from using a DEN-gas enhanced version of the CESI-MS the sequence has been organized as a standard profiling experiment using a block acquisition approach, where every block includes a random selection of the measured samples, QC samples and the analytical standards. CE-based metabolomics methods are usually criticized for low migration-time reproducibility^21^. Anticipating the problem, we took a special care on the alignment of the data using an in-house built tool, *msalign2*, which takes advantage of the superior mass accuracy of modern TOF instruments for migration-time correction^21^. Supplementary Fig. S-2 illustrates the effect of the alignment for measured QC samples (n = 11), where it can be seen that the variability of migration times was strongly reduced. We have also introduced a rigorous filtering step including in the analysis only the features which had a relative standard deviation (RSD) below 30% in the QC samples.

### Exploratory analysis of polycystic kidney disease tissue samples

**Figure 2:**
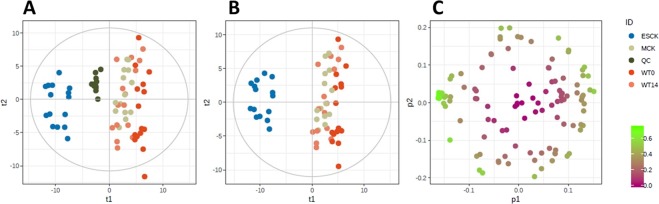
(**A**) PCA of log-transformed data. (**B**) PCA of data in (**A**), excluding QC samples. (**C**) PCA loading plot of 112 variables in (**B**), colored according to the modeling power of (**B**).

**Figure 3:**
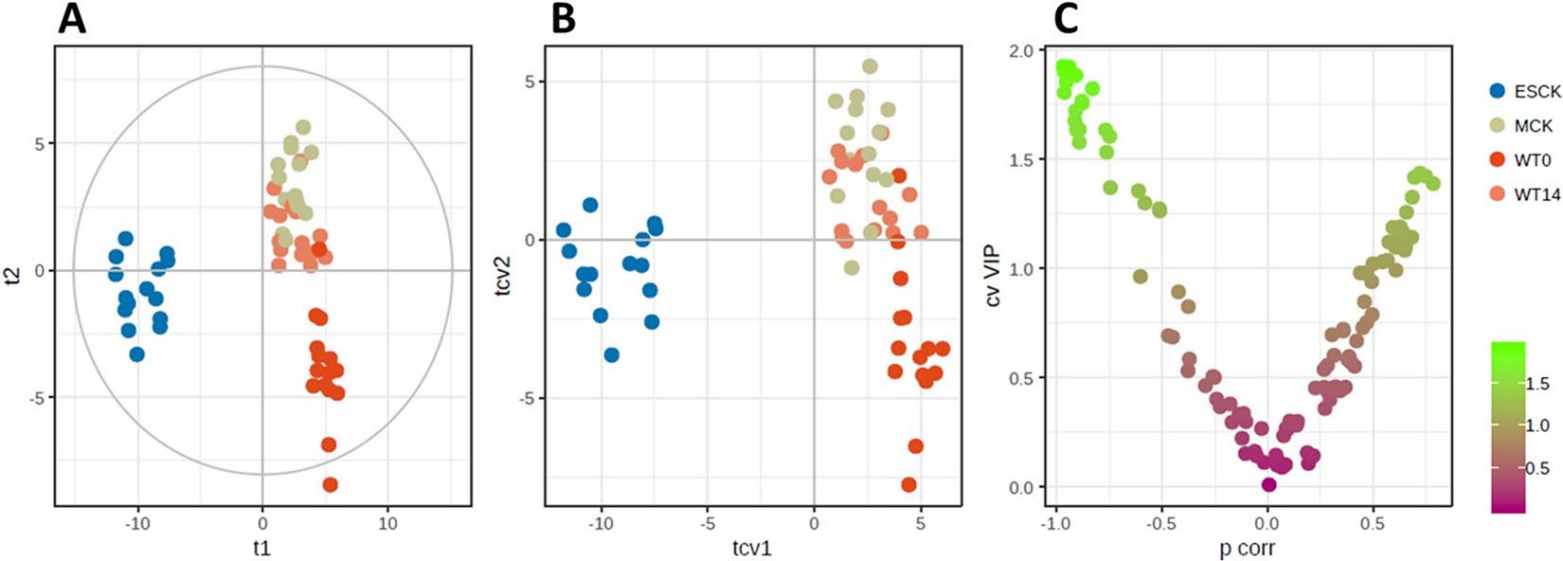
(**A**) PLS-DA score plot for the four groups of samples analyzed by the sheathless CE-MS platform. (**B**) Cross-validated score plot of the PLS-DA plot. (**C**) VIP *vs* p(corr) plot, colored according to the VIP values from (**B**).

The final data matrix after application of the 30% RSD filtering includes only 112 metabolic features. Even though the number of identified metabolic features seems rather low compared to other metabolomic studies^13^, it should be taken into account that the starting material was limited. In addition, the lower number in identification could be considered as an advantage, since data can be easily overfitted during multivariate modelling when limited samples are available (N = 60 mice). The number of detected metabolic features might have been enlarged if preconcentration approaches, e.g. dynamic pH junction^22^, were used. The essential first analysis step of the metabolic profiling experiment is evaluation of the analytical consistency and description of the main sources of the variance. The initial principal component analysis (PCA) modeling revealed a strong outlier - a MCK sample. Despite using probabilistic quotation normalization (PQN), abnormally low signal intensities were observed in this sample, indicating an analytical failure as a possible cause and consequently the sample was removed from further analysis. The score plot of the PCA model built after reprocessing the data without the outlier is presented in Fig. 2A. The model requires just three principal components to cover the first 50% of the variance, while the first two principal components covered 43% of the variance. The QC samples clustered together which indicates a proper analytical consistency and analytical variance within the limits expected for the CESI-MS method. It is worth mentioning that metabolic profiles for QC samples were not analyzed from multiple injections using the same injection vial, but were obtained from independent aliquots which had to be reconstituted in 2.5 µL of water every day before the start of the acquisition sequence. Figure 2B shows the score plot of the PCA model built without QC samples, similar characteristics were observed when compared to Fig. 2A, with only three components 50% of the variance was covered, whereas the first two components covered 44% of the variance. The score plot clearly shows that the end-stage cystic kidney (ESCK) group, which forms a distinct cluster, strongly influences the model. Figure 2C shows the loadings plot of the model presented in Fig. 2B. The plot is colored according to the modelling power emphasizing the cluster of the variables relevant for the model. As a next step we built a multi-class PLS-DA model using the experimental groups as the class ID. Figure 3A,B show the score plot and cross-validated score plot of the resulting model, respectively. The model parameters (R^2^X 0.75, R^2^Y 0.94, Q^2^ 0.5) and CV-ANOVA values (F = 13,1, p-value = 2.6 × 10^−30^) indicate a strong model. However, as well as in the PCA, samples from the ESCK group strongly influence the model, which complicates the interpretation of the VIP values (Fig. 3C). Apparently, the strongest ones will mainly explain the differences between the ESCK group and the remaining samples.

### Context-specific multivariate modelling

**Figure 4:**
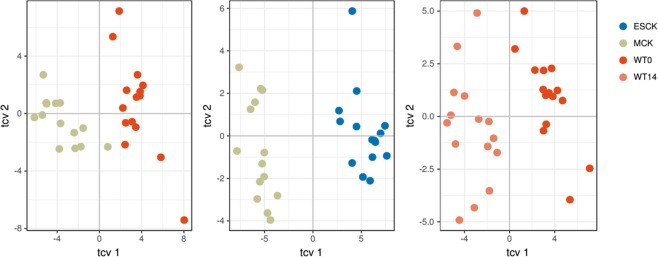
PLS-DA models for the three different pairwise comparisons performed in this work.

To get a more specific description of the metabolic changes between the groups, several two-class PLS-DA models were built, namely Wt0 (wildtype at timepoint 0) *vs* MCK (to explain the differences between non-PKD and mild PKD) and MCK *vs* ESCK (to explain a transition from mild to advanced stage of PKD). Finally, to get insight into metabolic changes associated with the aging of the animals, an additional model Wt0 *vs* Wt14 (Wt + 14 weeks) was built. Figure 4 shows the cross-validated score plots for all three models and Table 1 summarizes the model’s parameters. Despite the differences, all three models are valid. In agreement with the first two models (PCA and PLS-DA) the strongest model explained the differences between MCK and ESCK groups (highest values of R^2^X, R^2^Y, Q^2^ and F and the lowest p-value for CV-ANOVA from all models). From each model a subset of the most influential variables was selected by using the model specific variable importance on projection (VIP) values with an arbitrary cut-off of 1.35. Supplementary Fig. S-3 shows a Venn diagram summarizing all selected variables for the three models. Table 2 summarizes the features annotated according to the guidelines outlined in section 4.7.

**Table 1:** Number of latent variables, quality parameters (R^2^X, R^2^Y, Q^2^ and CV-ANOVA F and p-values) for the pairwise PLS-DA.

PLS-DA model	Number of latent variables	R^2^X	R^2^Y	Q^2^	CV-ANOVA	
					F	p-value
Wt0 *vs* MCK	3	0.440	0.987	0.923	35.0	3.6 × 10^−10^
MCK *vs* ESCK	3	0.569	0.988	0.967	95.0	1.4 × 10^−14^
Wt0 *vs* Wt14	2	0.311	0.969	0.857	37.8	2.9 × 10^−14^

**Table 2:** Annotation of variables with VIP values higher than 1.35 (highlighted in bold) in at least a pairwise PLS-DA model from the CE-MS metabolomics analysis of PKD samples.

Level of assignment	Annotation	Abbreviation	Migration time (min)	Detected specie	Experimental *m/z*	*m/z* error	VIP values of PLS-DA		
							Wt0 *vs* Wt14	Wt0 vs MCK	MCK *vs* ESCK
Confirmed with standard	Creatinine	Cre	13.4	[M+H]^+^	114.0671 115.0697	0.9 mDa	**2.03 1.75**	**1.56** 1.29	1.30 **1.36**
	Carnitine	Carn	15.4	[M+H]^+^	163.1159 (isotopic profile of 162.1139) 164.1173	1.4 mDa	0.37 0.31	0.91 0.95	**1.55 1.58**
	Creatine	Cr	17.0	[M+H]^+^	133.0796 (isotopic profile of 132.0782)	1.4 mDa	**1.49**	**1.42**	0.20
	Glutamine	Gln	21.0	[M-NH_3_+H]^+^	130.0503	0.5 mDa	1.27	**1.42**	0.59
	Betaine	Btn	22.9	[M+H]^+^	119.0899 (isotopic profile of 118.0884)	2.1 mDa	0.12	0.44	**1.39**
Tentative annotation	1-methylhistidine	mHis	14.6	[M+H]^+^	170.0925 171.0955	0.1 mDa	**2.31 2.29**	**2.17 2.20**	0.25 0.26
	Acetylcholine	Ach	14.7	[M+H]^+^	146.1178	0.2 mDa	0.91	**1.47**	0.72
	4-Guanidinobutanoic acid	Gba	15.0	[M+H]^+^	146.0927	0.3 mDa	1.04	**1.44**	**1.65**
	2-Aminooctanoic acid	AoA	15.2	[M+H]^+^	160.1334 161.1367	0.2 mDa	**1.53 1.54**	**1.81 1.86**	0.94 0.96
	Methylguanine (different isomers are possible)	mGu	15.2	[M+H]^+^	166.0725	0.2 mDa	0.46	0.61	**1.55**
	γ-L-Glutamylputrescine or N5-(1-imino-3-butenyl)-L-ornithine	Gput	15.4	[M-H_2_O+H]^+^	200.1393	0.6 mDa	**1.55**	**1.71**	1.01
	Formylisoglutamine	fGln	15.4	[M+H]^+^	175.0715	0.2 mDa	0.14	1.04	**1.60**
	Imidazolelactic acid	ILA	16.3	[M+H]^+^	157.0609	0.1 mDa	**1.66**	**1.66**	0.73
	Asn-Hydroxyproline or Hydroxyprolyl-Asn	P1	16.5	[M+H]^+^	246.1083	0.1 mDa	**1.41**	1.29	**1.57**
	Argininic acid	ArgA	16.5	[M+H]^+^	176.1030	0.0 mDa	0.20	0.19	**1.65**
	Propionyl-L-carnitine	Pcarn	16.6	[M+H]^+^	218.1386 219.1417	0.1 mDa	**1.36 1.37**	0.92 0.90	1.28 1.27
	Homocitrulline	Hcit	16.8	[M+H]^+^	190.1185	0.1 mDa	1.09	0.30	**1.64**
	Glycyl-lysine or lysyl-glycine	P2	17.4	[M+H]^+^	204.1342	0.1 mDa	0.68	0.28	**1.63**
	N-α-Acetyl-L-arginine	Aarg	17.7	[M+H]^+^	217.1286	0.9 mDa	**1.75**	0.82	**1.54**
	3-Hydroxyisovalerylcarnitine	HIVC	17.7	[M+H]^+^	262.1647	0.2 mDa	**2.07**	0.43	**1.47**
	(1) Phe-Thr or Thr-Phe (2) Hydroxyproline-Leu(Ile), or Leu(Ile)-Hydroxyproline	P3	18.5	[M+H]^+^ (1) [M+Na]^+^ (2)	267.1339 268.1370	0.9 mDa	**1.70 1.68**	1.57 1.86	**1.61 1.65**
	2-[3-carboxy-3-(methylammonio)propyl]-L-histidine	CMHis	19.0	[M+H]^+^	271.1400	0.1 mDa	0.52	1.27	**1.64**
	Proline betaine	ProB	23.1	[M+H]^+^	144.1023	0.4 mDa	**1.86**	**2.18**	**1.39**
	γ-carboxyglutamic acid	Gla	32.1	[M+H]^+^	192.0501	0.2 mDa	1.26	0.83	**1.35**
	5-Hydroxyindoleacetylglycine	hINaG	32.1	[M-H_2_O+H]^+^	231.0764	0.6 mDa	**1.88**	**1.67**	**1.56**
	Pantothenic acid	PA	32.2	[M-H_2_O+H]^+^	202.1074	0.5 mDa	0.06	0.15	**1.45**

### Biological Relevance of the Annotated Metabolites

**Figure 5:**
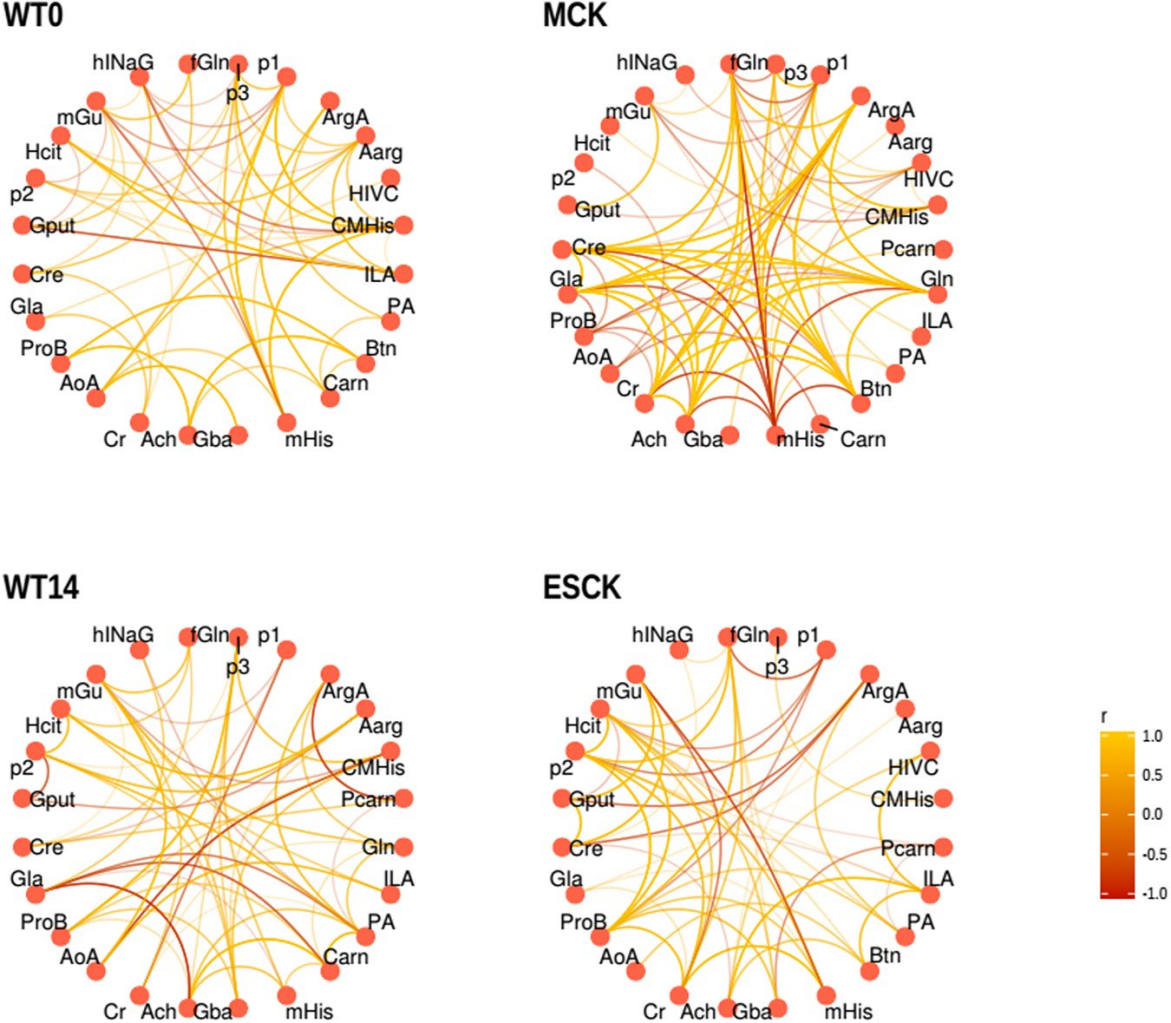
Correlational relationships between the annotated metabolites within each of the four experimental groups. For abbreviations see Table 2.

Table 2 summarizes all annotated features, however, the degree of confidence for the given annotations is varying. Figures S-4 and S-5 from supporting information display the box-plots for metabolites annotated unequivocally and tentatively, respectively. From this point the most logical question is to which degree the metabolites present in this table describe biology associated with the applied experimental design. There are several metabolites like e.g. creatinine, creatine, carnitine, and glutamine which have a proven track record of being associated with a renal physiology. The very fact that these metabolites are on the list illustrates that the selected analytical workflow is capable of targeting the relevant structures. Yet, verifying the results of a metabolomics experiment with the published data only provides hardly any novel information. Something that a post-genomics technology such as metabolomics is meant to do. Mapping the annotated structures to the biochemical pathways provides even less practical information. The flexibility of the mammalian biochemistry is such that a causal relation between the metabolites cannot be reconstructed from the profiling data without an experiment exploring a product-substrate relationship. To this end, we have explored an unbiased way of the data interpretation which uses the information available within the dataset: experimental design and the relative abundances of the metabolites. However, instead of a comparison between the experimental groups we have visualized the data in a form of the correlational relationships between metabolites within each experimental group. Figure 5 shows the results of such visualization. Notably, the most complex correlation structure at the given threshold of the inclusion (0.6) was observed within the MCK group. One of the “hubs” in this network is methylhistidine showing strong negative correlations to a number of the metabolites including creatine, creatinine, glutamine and a downstream product of the histidine metabolism, formylisoglutamine. It is interesting to note that the correlational structure of the MCK samples includes a number of the expected associations like e.g. creatine, creatinine, creatine-glutamine and some which till recent were not mentioned in the literature in the context of the renal physiology. It is important to keep in mind that the relationships visualized in Fig. 5 are not actual biochemical interactions but rather guidelines which can be used for follow-up research. To our opinion, creating the sets of the ideas for further validation in a more focused experimental design/setting is exactly the purpose of global metabolic profiling. Yet, one cannot ignore the fact that methylhistidine offers a few possibilities for speculation about its possible physiological role. For instance, the imidazole ring of methylhistidine was for long time considered as a prime source of the anti-oxidative activity of the histidine derivatives. Moreover, it has been shown that naturally occurring histidine containing the di-peptides carnosine and anserine have ability to chelate metals.

## Conclusions

Here, we present a first application of sheathless CE-MS (CESI-MS) for metabolic profiling of biomass-restricted tissue samples, namely individual sections of mouse kidney. After application of a strict data processing routine, consistent metabolic profiles were obtained covering more than 100 metabolic features, although minute amounts of sample were used (histological samples). Note that the number of metabolic features might have been increased if preconcentration strategies, such as dynamic pH junction, were used. The recorded metabolic profiles clearly differentiated the experimental groups included in this pilot study. Using a combination of non-supervised and supervised multivariate analysis we have dissected a subset of the metabolites underlying the differences between the experimental groups (non-PKD young mice, adult mice with PKD in mild state, adult mice with PKD in end-stage, and non-PKD adult mice). Several annotated compounds such as glutamine, carnitine, creatinine and creatine are well known for their roles in renal physiology. Using a data visualization procedure based on the correlational relationships between the metabolites, we could provide leads for further research. Overall, this study demonstrates the feasibility of sheathless CE-MS as a platform for metabolic profiling of biomass-restricted tissue samples, and it may open new possibilities for a better understanding of biological processes in sample-limited cases.

## Methods

### Chemicals and reagents

Sodium hydroxide (NaOH) was obtained from Merck (Darmstadt, Germany). Glacial acetic acid, water, hydrochloric acid (HCl), glutamine, carnitine, creatine, and creatinine were purchased from Sigma-Aldrich (Steinheim, Germany) and were of analytical grade or higher. Acetonitrile (MeCN) of LC–MS grade originated from Biosolve (Valkenswaard, The Netherlands). A standard metabolite mixture comprised of roughly 60 metabolites, with a concentration of 50 µM in water, was obtained from Human Metabolome Technologies (Tokyo, Japan), the so-called “HMT” mixture.

### Animals, Experimental Design and Sample Collection

Oral administration of tamoxifen was conducted at Post Natal (PN) days 40–42 to inducible kidney-specific *PKD1*-deletion mice (tam-KspCad-CreERT2;PKD1^lox2–11;lox2–11^ mice) as previously reported^23^. At 8–12 weeks after gene disruption a subgroup of mice was sacrificed (MCK group: mild cystic kidneys). These mice displayed tubular dilations and small cysts accompanied by increased 2KW/BW% (1.9–3.3%). Another group of mice was sacrificed at renal failure (ESCK group: end stage cystic kidneys) having large 2KW/BW% (11–17%). A blood urea concentration of ≥20 mmol/L was used as an indicative of renal failure. In addition, there were two control group of mice having normal kidneys and not receiving tamoxifen, mice of age PN40 (Wt0) and at age of PN40 + 14 weeks (Wt14). Therefore, the study was composed of a total of four groups (Wt0, Wt14, MCK, and ESCK) having five mice each, being twenty the total number of subjects.

Snap-frozen kidneys were attached to a cryotome table using the embedding medium for frozen tissue KP-CryoCompound (Klinipath BV, The Netherlands). Three 20 µm-thick sections of the same kidney were obtained per mouse. Sections were collected in 1.5 mL microtubes and they were kept at −80 °C until sample preparation was conducted.

All the animal experiments were approved by the Animal Experiment Ethics Committee of Leiden University Medical Center and the Commission Biotechnology in Animals of the Dutch Ministry of Agriculture and all the methods were performed in accordance with their guidelines and regulations.

### Sample preparation

Sample preparation was a continuation of the work by Sánchez-López *et al*. in which the samples were analyzed on liquid chromatography-MS platforms (both RPLC, and HILIC)^24^. Upon evaporation and reconstitution of the samples in 80:20 (MeCN:water, v/v), 7.5 µL were evaporated for 15 min at room temperature in a SpeedVac instrument (Eppendorf, model 5301) and reconstituted in 2.5 µL of water for sheathless porous tip CE-MS analysis. QC pool for the CE-MS platform was prepared by taking 1 µL of each sample (N = 20) and mixing thoroughly.

### Sheathless CE-MS conditions

CE analyses were performed on a Sciex/Beckman Coulter CESI 8000 system (Sciex, Framingham, MA) equipped with a temperature-controlled sample tray, kept at 10 °C, and a power supply able to deliver up to 30 kV. Separation was performed using a bare fused silica capillary (91 cm × 30 μm i.d. × 150 μm o.d.) and carried out by applying a voltage of 20 kV without any pressure applied. Prior to each sample injection, the capillary was rinsed with 0.1 M NaOH for 2.5 min, water for 4 min, 0.1 M HCl for 2.5 min, water for 4 min, and background electrolyte (BGE) for 4 min. The BGE was a solution of 10% (*v/v*) acetic acid (pH 2.3). All samples were hydrodynamically injected applying a pressure of 1 psi for 60 s (which corresponds to 1.4% of the capillary volume, injecting 9 nL). A plug of BGE was injected after the sample injection by applying a pressure of 0.5 psi for 25 s (corresponding to 0.3% of the capillary volume). CE vials were built “in-house” by manually cutting the tip of a PCR microtube and introducing it in a Sciex/Beckman Coulter 1.5 mL vial by means of a stainless spring (see Fig. S-6). This setup enabled working with vial volumes as low as 2.5 µL per sample.

The CE apparatus was coupled to a UHR-QqTOF maXis Impact HD mass spectrometer from Bruker Daltonics (Bremen, Germany) via a sheathless porous tip CE-MS interface based on a custom-made platform from Sciex/Beckman Coulter (Brea, CA) allowing for an optimal position of the capillary porous tip in front of the MS nanospray shield (Bruker Daltonics). Dopant enriched nitrogen (DEN)-gas at 0.2 bar was used along the sequence. To this end, an in-house made polymer cone was slid onto the housing of the porous tip allowing for a coaxial sheath flow of the DEN-gas around the ESI emitter. The concentration of acetonitrile in the DEN-gas corresponds to ∼4% (mole percentage). All CE-MS experiments were carried out in ESI positive mode with a capillary voltage of 1,000 V. Drying gas flow (N_2_) rate was 1.2 L/min at 150 °C. MS data was acquired between *m/z* 50 and 1,300 with a spectral acquisition rate of 1 Hz. For MS/MS experiments, all parameters were similar to the ones for MS, except for the spectra acquisition that was registered from *m/z* 30 to 1,200. Collision energy was set to 20 V. MS and MS/MS data were acquired with DataAnalysis 4.2 (Build 387, Bruker Daltonics).

### CE-MS metabolomics sequence

At the beginning of the metabolic profiling sequence, blanks, QC pools and standard metabolite mixtures from Human Metabolome Technologies (HMT) were injected in the CE-MS platform. Samples of PKD study were randomized for injection, and a QC pool was injected after seven samples were injected. The HMT mixture was injected at least once per day to assess the metabolite annotation (the metabolomic sequence lasted for 5 days).

### Data treatment and data analysis

MS data files obtained by the CE-MS platforms were recalibrated based on sodium acetate clusters and were exported in the.mzXML format. Alignment was performed by an in-house tool, *msalign2*^21^. Peak picking was carried out on the aligned data using XCMS R-package (The Scripps Research Institute, La Jolla, CA) based on the centWave algorithm using the following settings: maximum tolerated *m/z* deviation in consecutive scans, 15 ppm; chromatographic peak width, 5–25 s; scan range, 60–2325 s; scan range to process, *m/z* 50–1,300; noise, 20,000; prefilter step, at least 3 peaks with intensity > 50,000; *m/z* center of the feature, wMean (intensity weighted mean of the feature *m/z* values); signal-to-noise ratio threshold, 50; minimum difference in *m/z* for peaks with overlapping migration time, 0.01 min. After peak picking, peak grouping was performed with the following parameters: bandwidth, 2; and *m/z* width of 0.01. After peak picking and grouping, variables having RSD values above 30% in the QC were excluded.

Before multivariate analysis, the data was normalized using the PQN algorithm. PQN is based on the determination of the most probable dilution factor by considering the distribution of the quotients of the signals of a certain sample by those of the QC samples^25^. Multivariate statistical analysis was carried out by means of SIMCA 14 (Umetrics, Umeå, Sweden).

Base peak electropherogram and extracted ion electropherogram Figures were plotted using MZmine 2^26^. The data visualization was performed using R version 3.4 and packages, “Rcpm”, “ggplot”, “igraph”, “ggraph”, “corrr”. For Fig. 5 the data set was divided according to the conditions MCK, ESCK, Wt0, Wt14, and a correlation matrix was built on every subset separately and converted into a long format; this output was filtered to retain only the correlations with absolute value above 0.6 and passed to the “ggraph” function using “circular” outline for visualization.

### Metabolite annotation

Highly influencing variables in the PLS-DA models were tentatively identified by looking up their *m/z* values in different databases (METLIN (http://metlin.scripps.edu), HMDB (http://hmdb.ca), and KEGG (http://genome.jp/kegg)), establishing 20 ppm as the error width. The identity of carnitine, glutamine, creatinine, betaine and creatine could be confirmed by matching the migration time and MS/MS spectra pattern obtained from standards. Tentative identities were annotated based on databases.

## Supplementary information

Supplementary Information

Supplementary information - Dataset

## Data Availability

The dataset generated and analyzed during this study is included in the Supplementary Information file of this article.
