# Supplementary material for: Sheathless CE-MS based metabolic profiling of kidney tissue section samples from a mouse model of Polycystic Kidney Disease
Source: Sci Rep. 2019 Jan 28;9:806. doi: 10.1038/s41598-018-37512-8 (PMC6349881; doi:10.1038/s41598-018-37512-8)
Supplement: Supplementary file 1 — Supplementary Information [file 41598_2018_37512_MOESM1_ESM.docx]

**Scientific Reports**

**Electronic Supplementary Material**

**Sheathless CE-MS based metabolic profiling of kidney tissue section samples from a mouse model of Polycystic Kidney Disease**

Elena Sánchez López*^1^*, Guinevere S.M. Kammeijer*^2^*, Antonio L. Crego^1^, María Luisa Marina^1^, Rawi Ramautar*^3^*, Dorien J. M. Peters^4^, Oleg A. Mayboroda*^2*^*

*^1^ Department of Analytical Chemistry, Physical Chemistry and Chemical Engineering, University of Alcalá, Madrid, Spain*

*^2^ Center for Proteomics and Metabolomics, Leiden University Medical Center, Leiden, The Netherlands*

*^3^ Biomedical Microscale Analytics, Leiden Academic Center for Drug Research, Leiden University, The Netherlands*

*^4^ Department of Human Genetics, Leiden University Medical Center, Leiden, The Netherlands*

**^*^Correspondence**: Oleg A. Mayboroda, Leiden University Medical Center, Center for Proteomics and Metabolomics, P.O. Box 9600, 2300 RC Leiden, The Netherlands; o.a.mayboroda@lumc.nl; Tel: +31-71-52-66395

**Table of contents:**

[Figure S-1 3](#_Toc515014275)

[Figure S-2 4](#_Toc515014276)

[Figure S-3 5](#_Toc515014277)

[Figure S-4 6](#_Toc515014277)

[Figure S-5 7](#_Toc515014277)

[Figure S-6 9](#_Toc515014277)

# Figure S-1

**
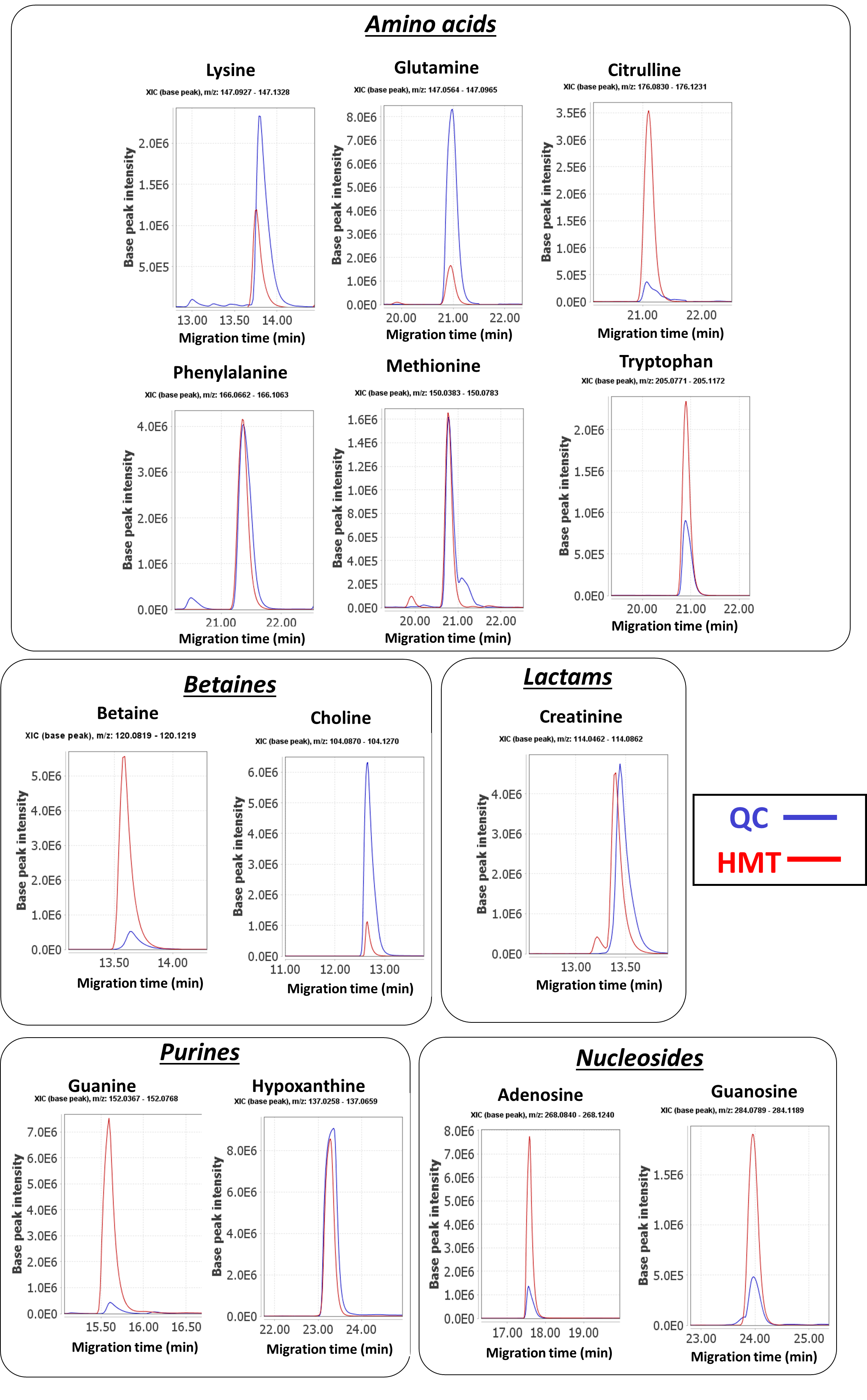
**

**Figure S-1** Extracted ion electropherograms obtained for a selection of amino acids, betaines, lactams, purines and nucleosides which were detected both in the QC (blue line) and in the standard human metabolite mixture (HMT; red line) samples by sheathless CE-MS. All standards in the metabolite mixture had a concentration of 50 µM.

# Figure S-2

**
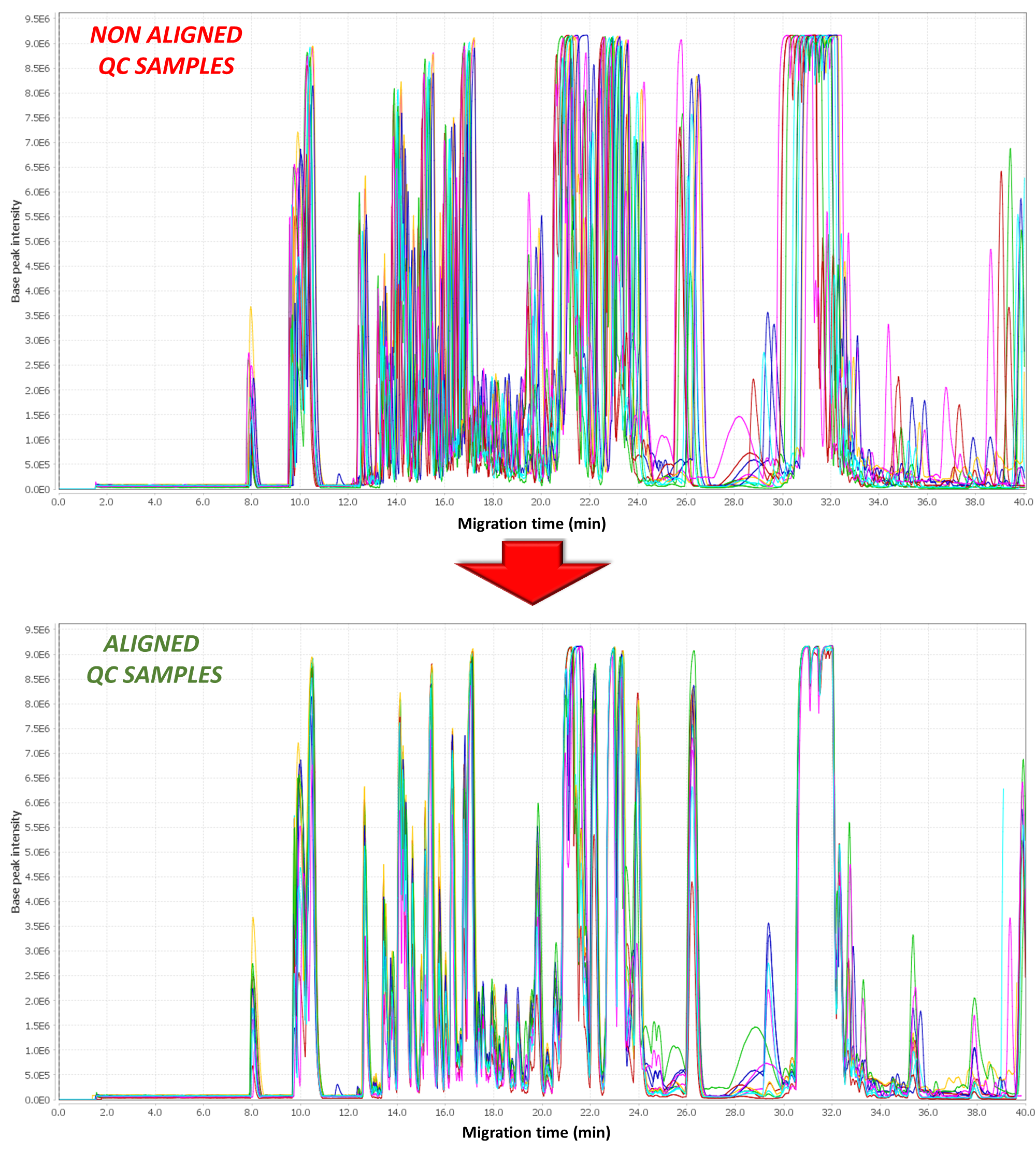
**

**Figure S-2** Overlapped base peak electropherograms of QC samples (N=11) before and after migration time alignment.

# Figure S-3

**
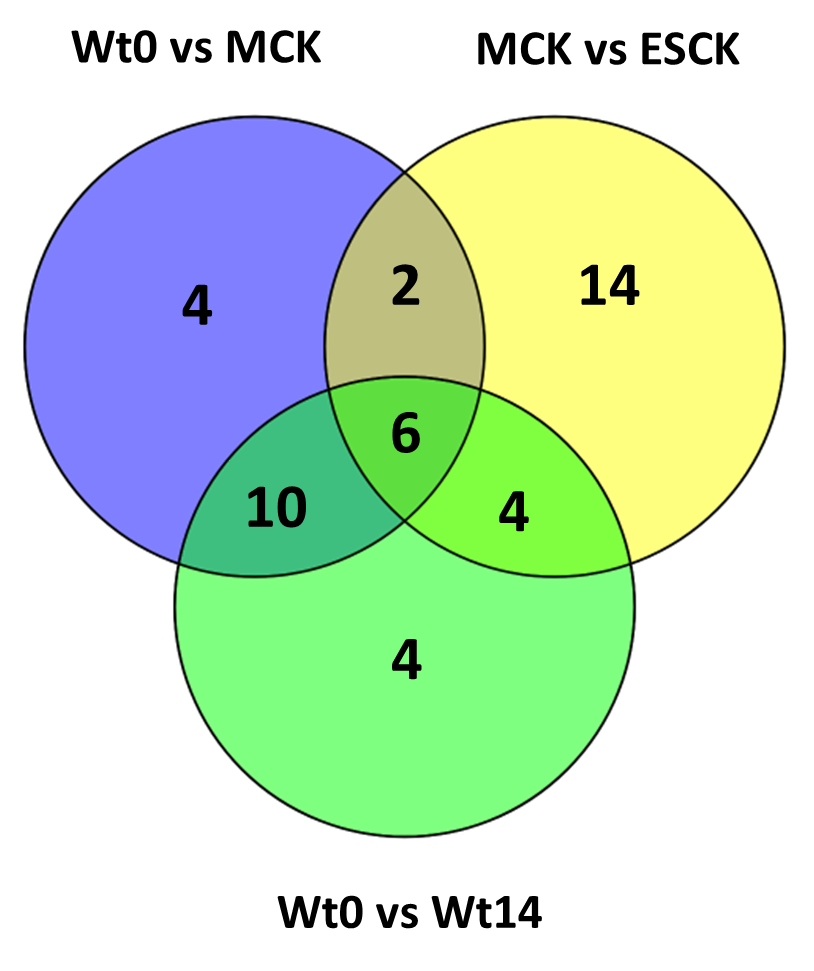
**

**Figure S-3** Venn diagram displaying the number of variables with VIP higher than 1.35 for each of the pairwise PLS-DA models from this work. Wt0 and Wt14 represents the wild type groups at time point zero and 14 weeks, respectively. MCK and ESCK represents groups with mild kidney damage conditions and end-stage kidney damage conditions, respectively.

# Figure S-4


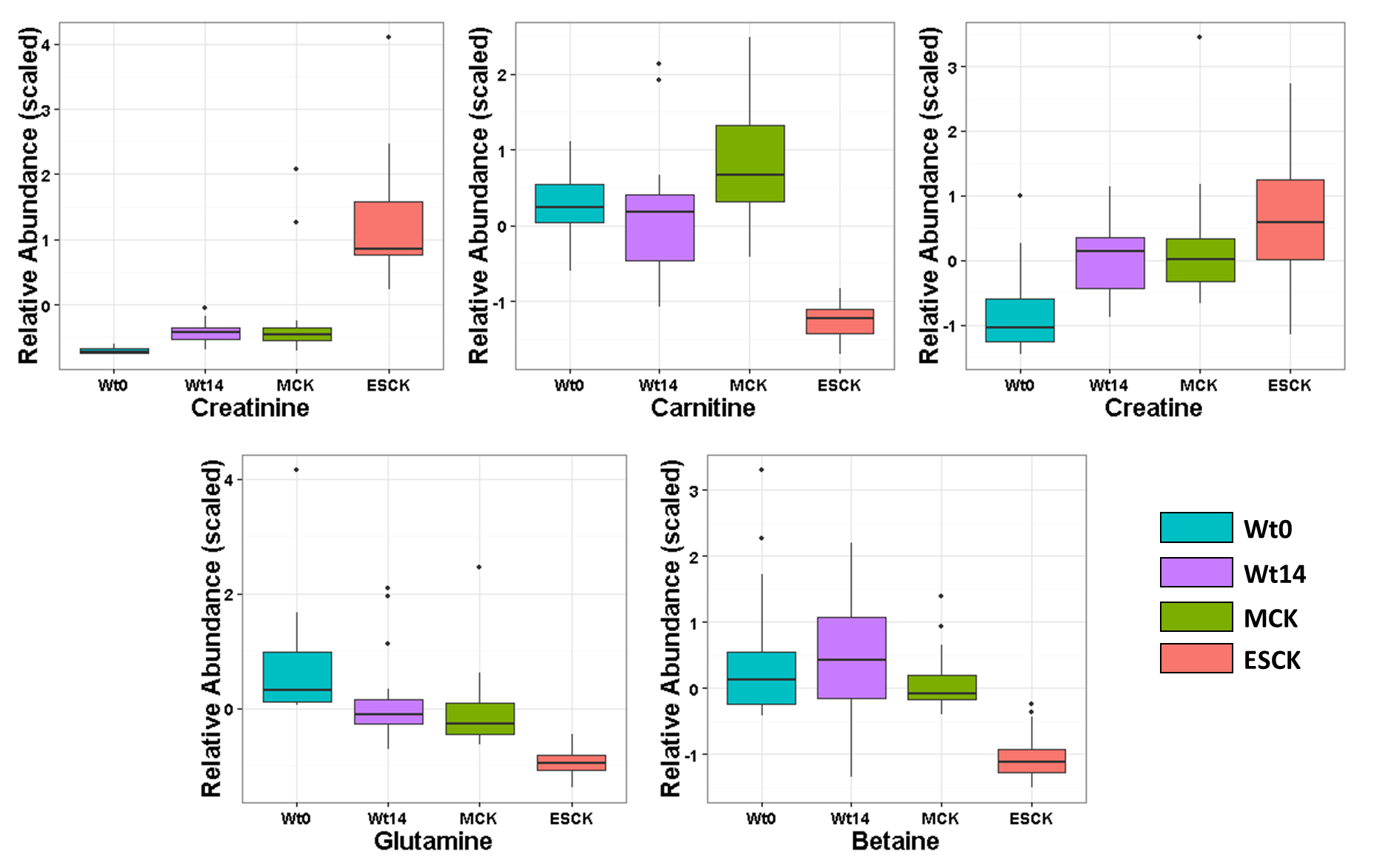


**Figure S-4** Box-plots for metabolites unequivocally identified. Wt0 and Wt14 represents the wild type groups at time point zero and 14 weeks, respectively. MCK and ESCK represents groups with mild kidney damage conditions and end-stage kidney damage conditions, respectively.

# Figure S-5

**
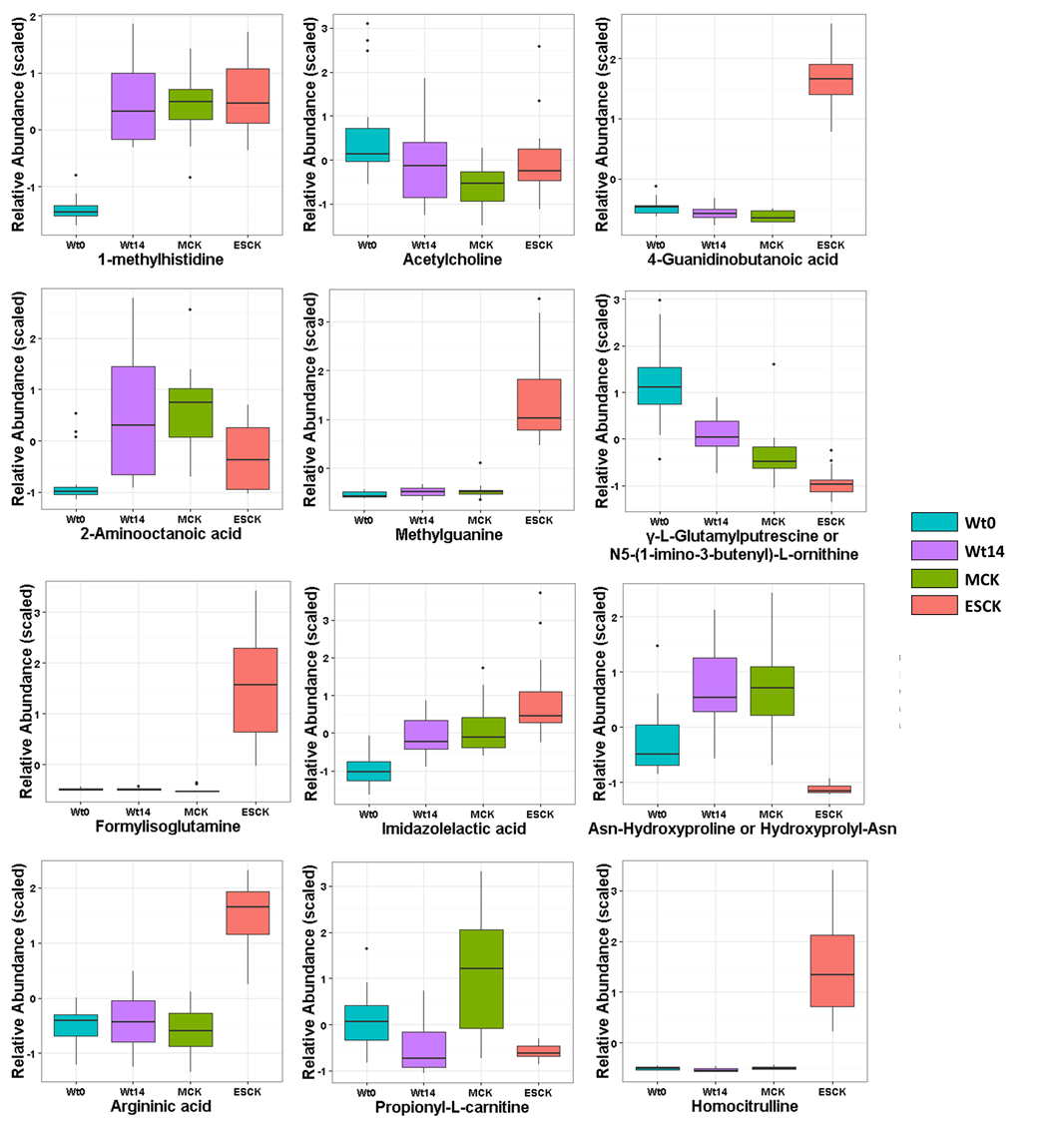
**

# Figure S-5 (continuation)

**
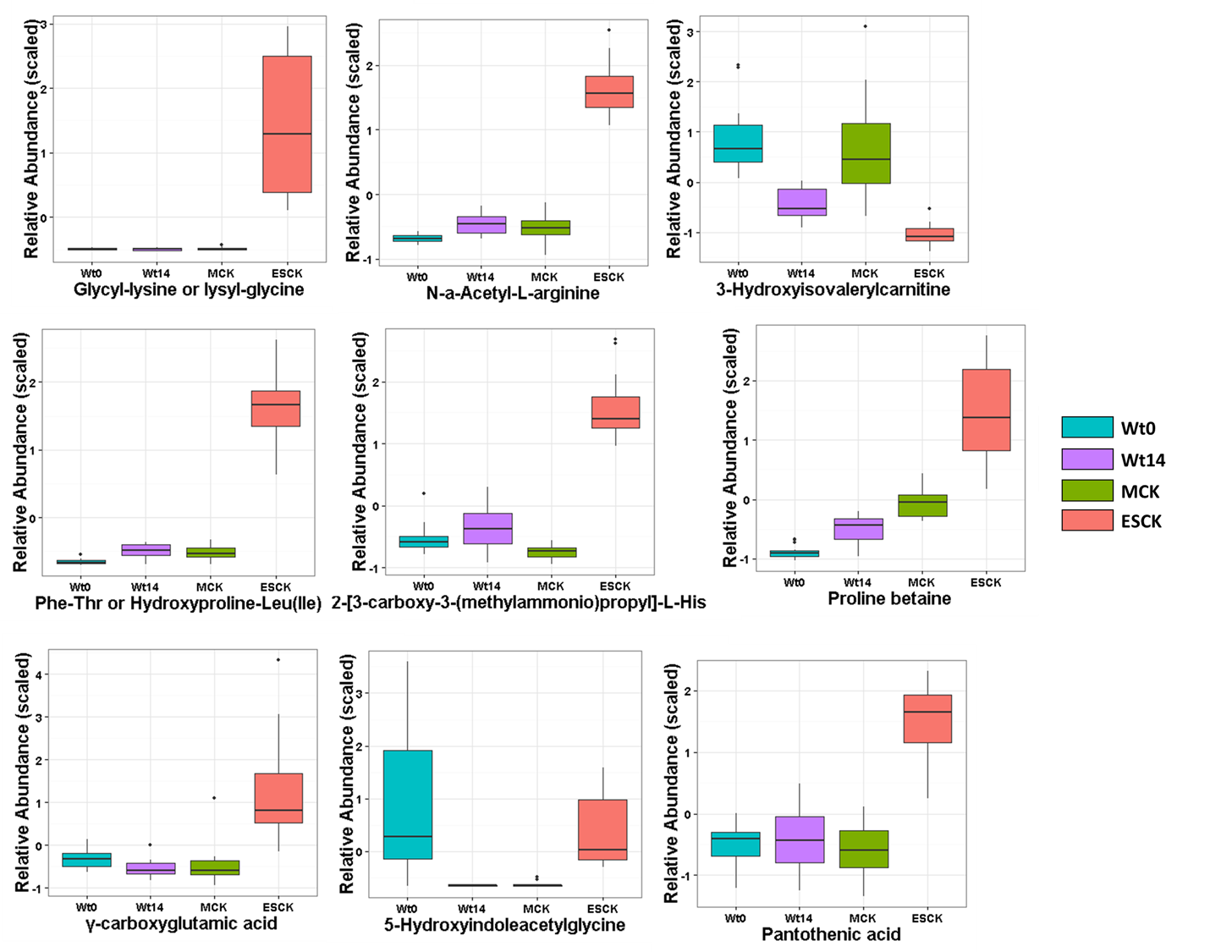
**

**Figure S-5** Box-plots for metabolites tentatively identified. Wt0 and Wt14 represents the wild type groups at time point zero and 14 weeks, respectively. MCK and ESCK represents groups with mild kidney damage conditions and end-stage kidney damage conditions, respectively.

# Figure S-6

**Figure S-6** Illustration of the in-house vial set-up that was used in this study.
